# Evaluating spatial disparities of rotor sites and high dominant frequency regions during catheter ablation for PersAF patients targeting high dominant frequency sites using non-contacting mapping

**DOI:** 10.3389/fphys.2022.946718

**Published:** 2022-08-05

**Authors:** Mahmoud Ehnesh, Xin Li, Tiago P. Almeida, Gavin S. Chu, Nawshin Dastagir, Peter J. Stafford, G. André Ng, Fernando S. Schlindwein

**Affiliations:** ^1^ School of Engineering, University of Leicester, Leicester, United Kingdom; ^2^ Department of Cardiovascular Sciences, Glenfield Hospital, Leicester, United Kingdom; ^3^ National Institute for Health Research Leicester Cardiovascular Biomedical Research Centre, Leicester, United Kingdom; ^4^ Department of International Foundation, Massey University, Auckland, New Zealand

**Keywords:** atrial fibrillation, cycle length, high dominant frequency, non-contact mapping, rotors

## Abstract

**Purpose:** Several studies have emphasised the significance of high dominant frequency (HDF) and rotors in the perpetuation of AF. However, the co-localisation relationship between both attributes is not completely understood yet. In this study, we aim to evaluate the spatial distributions of HDF regions and rotor sites within the left atrium (LA) pre and post HDF-guided ablation in PersAF.

**Methods:** This study involved 10 PersAF patients undergoing catheter ablation targeting HDF regions in the LA. 2048-channels of atrial electrograms (AEG) were collected pre- and post-ablation using a non-contact array (EnSite, Abbott). The dominant frequency (DF, 4–10 Hz) areas with DF within 0.25 Hz of the maximum out of the 2048 points were defined as “high” DF (HDF). Rotors were defined as PSs that last more than 100 ms and at a similar location through subsequent phase frames over time.

**Results:** The results indicated an extremely poor spatial correlation between the HDF regions and sites of the rotors in pre-versus post-ablation cases for the non-terminated (pre: CORR; 0.05 ± 0.17. vs. post: CORR; −0.030 ± 0.19, and with terminated patients (pre: CORR; −0.016 ± 0.03. post: CORR; −0.022 ± 0.04). Rotors associated with AF terminations had a long-lasting life-span post-ablation (non-terminated vs. terminated 120.7 ± 6.5 ms vs. 139.9 ± 39.8 ms), high core velocity (1.35 ± 1.3 mm/ms vs. 1.32 ± 0.9 mm/ms), and were less meandering (3.4 ± 3.04 mm vs. 1.5 ± 1.2 mm). Although the results suggest a poor spatial overlapping between rotors’ sites and sites of AFCL changes in terminated and non-terminated patients, a higher correlation was determined in terminated patients (spatial overlapping percentage pre: 25 ± 4.2% vs. 17 ± 3.8% vs. post: 8 ± 4.2% vs. 3.7 ± 1.7% *p* < 0.05, respectively).

**Conclusion:** Using non-contact AEG, it was noted that the correlation is poor between the spatial distribution of HDF regions and sites of rotors. Rotors were longer-lasting, faster and more stationary in patients with AF termination post-ablation. Rotors sites demonstrated poor spatial overlapping with sites of AFCL changes that lead to AF termination.

## Introduction

Atrial fibrillation (AF) is the most common arrhythmia, with a fivefold increased risk of stroke (Nattel, 2002) and affecting more than 10% of people aged 65 and over (Freedman et al., 2021).

Catheter ablation proved to be effective for Paroxysmal AF (PAF) (Fichtner et al., 2015), however, as persistent AF (PersAF) is a more complex arrhythmia, it is more difficult to treat (Jalife et al., 2002; Nattel, 2002).

Studies have shown electrically remodelled tissue can potentially host rapid ectopic activity, and single and multiple circuit re-entry, perpetuating AF (Jalife et al., 2002; Nattel, 2002). Atrial electrograms (AEG) characterised by short cycle length (CL) and high-frequency activation (Mansour et al., 2001; Ashihara et al., 2012) have been proposed to identify local abnormal activations during AF (Mansour et al., 2001). Studies suggested that AEG with high-dominant frequencies (HDF) represent atrial regions with rapid ectopic activity (Sanders et al., 2005; Jarman et al., 2012) and these regions have been proposed as potential targets for AF ablation (Atienza et al., 2009). Their ablation resulted in CL prolongation and AF termination (Sanders et al., 2005). However, others argue that it remains unclear whether these sites are spatiotemporally stable (Habel et al., 2010; Jarman et al., 2012; Atienza et al., 2014).

Narayan et al., 2012; Spitzer et al., 2017 proposed targeting re-entry circuits and focal impulse for treating AF, other researchers reported contradictory findings (Balouch et al., 2017; Steinberg et al., 2017) due to rotors being spatiotemporally unstable, suggesting that these rotors may not be associated with AF perpetuation (Yamabe et al., 2016).

The co-localisation relationship study between DF and phase singularity points (PSs) in AEG has revealed that most PSs are located near DF areas, suggesting that wave breaks occur close to those boundaries (Samie et al., 2001; Salinet et al., 2017). Whereas with respect to non-contact AEG (NC-AEG), Salinet et al. (2017), reported that clusters of paired PSs spatially correlate with DF regions. Therefore, identifying ablation targets on NC-AEG using spectral analysis or phase mapping has not been fully understood yet.

AF cycle length (AFCL) has been shown to reflect local atrial refractoriness in animals and humans and has been suggested as a parameter to assess the effectiveness of AF ablation (O'Neill et al., 2009; Rostock et al., 2011). Thus, the study of the co-localisation relationship between HDF regions and rotors (re-entrant activity) and its role in perpetuating AF is important. No study to date has evaluated the spatial correlation between rotor sites and sites of short AFCL that lead to AF termination in NC-AEG.

The present study aims to evaluate 1) the spatial distributions of HDF regions and rotor sites within LA pre-/post- HDF-guided ablation in PersAF, 2) the response of the rotors to HDF-guided ablation, 3) the spatial correlation between rotor sites and sites of AFCL changes that lead to AF termination.

## Materials and methods

### Electrophysiology study

Ethical approval was secured for the present study from the local ethical committee at the University Hospitals of Leicester, National Health Service (NHS) Trust. Their guidelines were ascribed to and followed accordingly. Ten patients undergoing catheter ablation of PersAF for the first time, with no previous heart disease, were recruited (Bonow et al., 2006). All individuals were in AF at the time the procedure commenced. All antiarrhythmic medications aside from amiodarone were discontinued 5 days prior to the procedure. Routine blood sampling and a twelve-lead of electrocardiogram (ECG) were collected from all participating patients.

### Non-contacting mapping

**FIGURE 1:**
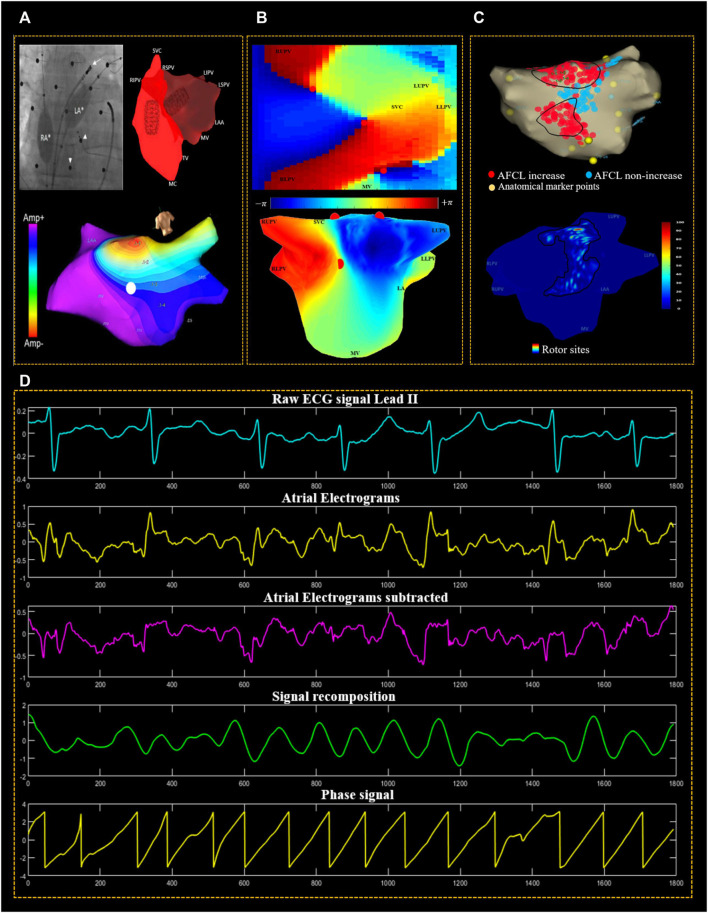
The methodological procedure used for data acquisition and signal processing. **(A)** Fluoroscopic image of the non-contact balloon catheters deployed in both the left atrium (LA) and right atrium (RA) (white asterisks). The small white arrows heads point at the active electrodes of the body surface mapping (BSM), and the white arrow points at the ablation catheter. The screenshot of the EnSite velocity mapping system illustrates the LA geometry isopotential/voltage map. **(B)** Reconstructed 2D & 3D of left atrial geometry with a colour-coded phase map; the colour bar indicates that the phase varies from −π to +π and the colour coding represents this 2π phase range from blue to red. PSs are represented by red circles. **(C)** LA Geometry exported from EnSite non-contact mapping system, showing regions in the LA where ablations resulted in AFCL increase (red), AFCL non-increase (blue). Anatomical marker points are indicated by (yellow) dots; and 3D geometry of LA was constructed using in-house software USURP-AF (Understanding the electrophysiological SUbstRate of Persistent Atrial Fibrillation). The colour of the LA 3D geometry indicates a histogram of regions that host rotors, with a colour gradient of red to blue representing the percentage of the most visited to the less often visited region. **(D)** Phase mapping used reconstructed non-contact atrial electrograms (AEG). The aqua signal is the surface ECG (lead II) used as a reference for QRST subtraction of atrial electrograms. The second (yellow) signal is the unipolar AEG. Third, (magenta signal) is the QRST subtracted AEG, fourth (green signal) is the recomposed signal using sinusoidal wavelet reconstruction and fifth, the phase signal (yellow) is found using Hilbert transform.

Under local anaesthetic, an ablation catheter was introduced *via* the femoral vein into the right atrium (RA). Then, with fluoroscopic guidance, an inflatable decapolar catheter and a quadripolar catheter were positioned in the coronary sinus (CS) and his position, respectively. For all cases, a single transeptal puncture technique was employed to provide access into the patients’ left atrium (LA). This was realised by using a non-steerable transeptal sheath channel (Channel sheath, Bard Electrophysiology, United States). Heparin anticoagulation was administered to maintain an activated clotting time >300 s. Using a non-contact mapping (NCM) system (EnSite, Abbott Laboratories, United States) with multiple electrode array (MEA), high-resolution 3D geometries were reconstructed for the LA and RA, using the EnSite Velocity electro-anatomical mapping system (Abbott laboratories; Figure 1A). Using the non-contact MEA, 5-min intervals of intracardiac AEG were simultaneously recorded in AF for 2048 points.

### Radiofrequency ablation

Identification of the HDF regions was undertaken for ablation as described in (Salinet et al., 2014). During the ablation, certain criteria were followed to evaluate the ablation response; after ablating each region of HDF, the ablation catheter was used to measure AFCL change in the left atrium appendage (LAA) over 10 cycles. A change in AFCL by 10 ms or more was considered significant (Bezerra et al., 2020). These steps were repeated until one of the pre-determined endpoints was met:

• Conversion of AF to sinus rhythm (SR);
• Conversion of AF to a more organised LA rhythm, or;
• The operator decided to stop the ablation due to a lack of target coverage or for patient safety concerns.

Further AEG recordings were collected post-procedure for another 5 minutes. After the MEA was extracted, a standard Pulmonary Veins isolation (PVI) was performed targeting the Ostia of the Pulmonary Veins (PVs), irrespective of the atrial rhythm. Multipolar Pulmonary Vein Ablation Catheter (PVAC) (Medtronic, Fridley, MN, United States) was used for this ablation in all patients. Four out of the 10 patients had AF termination: One converted to SR and three progressed to atrial Flutter (AFL). There were no adverse events in any of the enrolled patients.

### Data collection of atrial electrograms and atrial fibrillation cycle length measurement

A non-contact MEA balloon was used for all patients (EnSite Velocity, Abbott Laboratories, United States). This device collected 2048 points of AF AEG from the endocardial surface of the LA over a period of 5 minutes. These were collected with a sampling frequency of 2034.5 Hz and subsequently exported using a 1–150 Hz band-pass filter set by the Ensite system. The Surface ECG was band-pass filtered (0.5–50 Hz). The collected data were analysed off-line using a MATLAB (MathWorks, United States) in-house developed program. Next, the AEG were resampled at 512 Hz using a cubic spline interpolation technique to reduce the processing time. This is noteworthy because the AEG were primarily sampled at 2034.5 Hz. Down-sampling the electrograms to 512 Hz does not result in loss of information in the AEG (Stevenson and Soejima, 2005). QRST subtraction was performed on the AEG to remove the far-field ventricular influence using the technique previously described in (Salinet et al., 2013). The bipolar signal at the LAA is clearly defined, allowing for an unambiguous manual assessment of AFCL, which has been used as a surrogate for AF organisation in several clinical studies (Haïssaguerre et al., 2004; Rostock et al., 2011; Honarbakhsh et al., 2018a). In this study, the AFCL in LAA was manually measured pre-and post- HDF-guided ablation using the ablation catheter over ten cycles to evaluate ablation response at the ablated site with a ±10 ms change considered significant (Figures 1B, C) (Bezerra et al., 2020).

### Frequency domain analysis of the atrial electrograms

The NC-AEG of 60 s-long recordings were then separated into 4 s windows, with an overlap of 2 s. Accordingly, the spectral resolution was 0.25 Hz. Fivefold zero-padding was used and applied to improve the identification of spectral peaks with a frequency step of 0.05 Hz. DF for each of the 2048 points was defined as the highest power between 4–10 Hz. To measure the regularity and variability of AEG in the spectrum, the regularity index (RI) and organisation index (OI) of each DF were calculated. The RI is calculated by dividing the area under the DF peak and its harmonics by the entire spectra. The OI was calculated as the ratio of the area under the curve of the DF peak to the total power of the spectrum (Tuan et al., 2010). For more information regarding the pre-processing steps of NC-AEG see Section 2.4.

### Identification of the high dominant frequency regions

**FIGURE 2:**
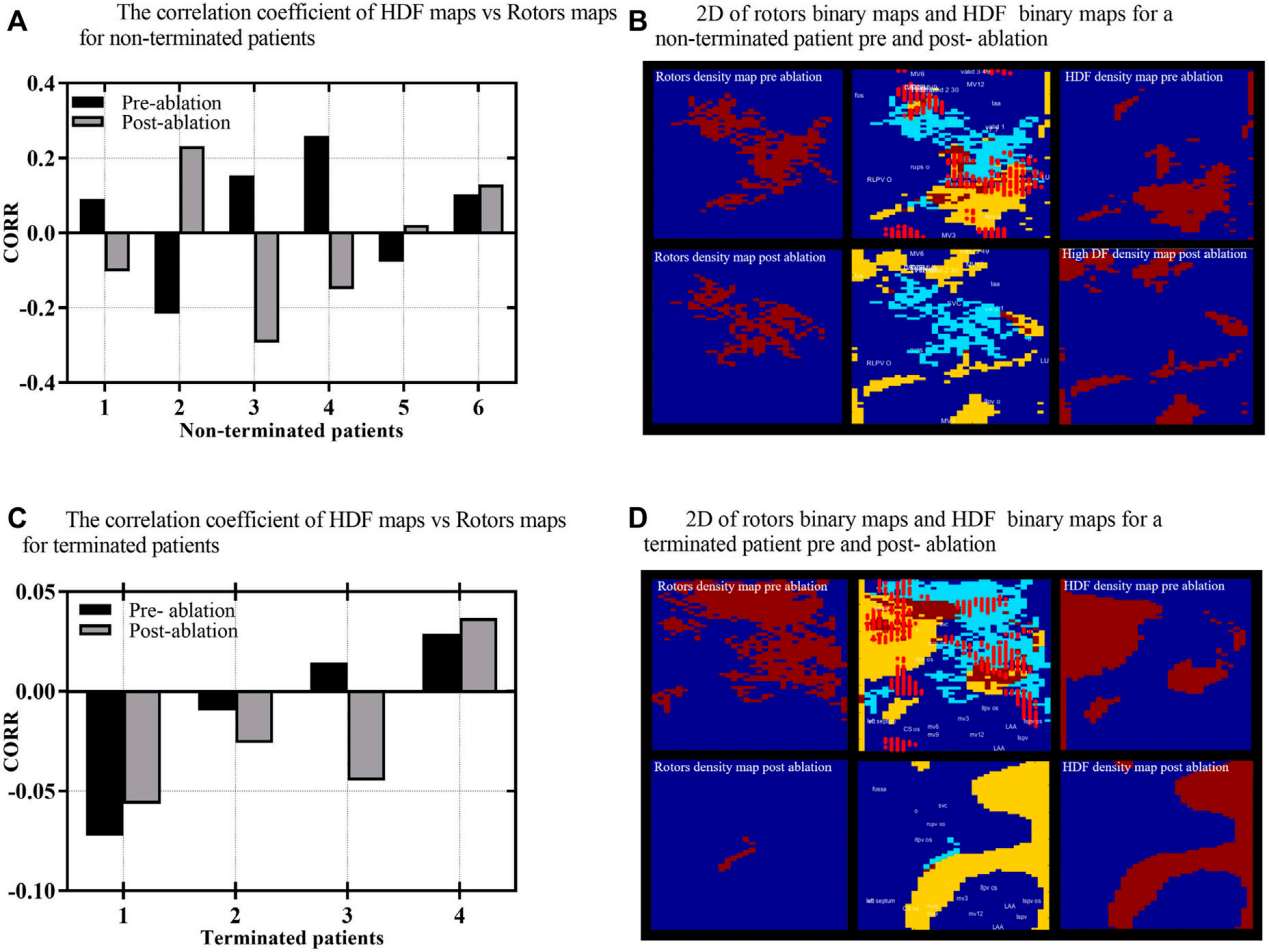
**(A)** The correlation coefficient (CORR) of HDF binary maps versus rotors binary maps for non-terminated. **(B)** 2D rotors binary maps on the right and HDF-binary maps on the left for non-terminated patient pre-and post-ablation. In the middle is a combined map of rotor binary map and HDF-binary map. **(C)** The CORR of HDF binary maps versus rotors binary maps for terminated patients pre-and post-ablation, respectively. **(D)** 2D rotors binary maps on the right and HDF-binary maps on the left for terminated patient pre- and post-ablation; in the middle is a combined map of rotor binary map and HDF-binary map. The yellow colour in combined maps indicates the HDF region, light blue indicates rotors, with the brown colour indicating the overlapping area, and the bright red dots represent ablation lesions marked by the mapping system during the procedure. The other maps are binary with brown indicating either HDF or rotors and blue representing areas that do not host either.

**FIGURE 3:**
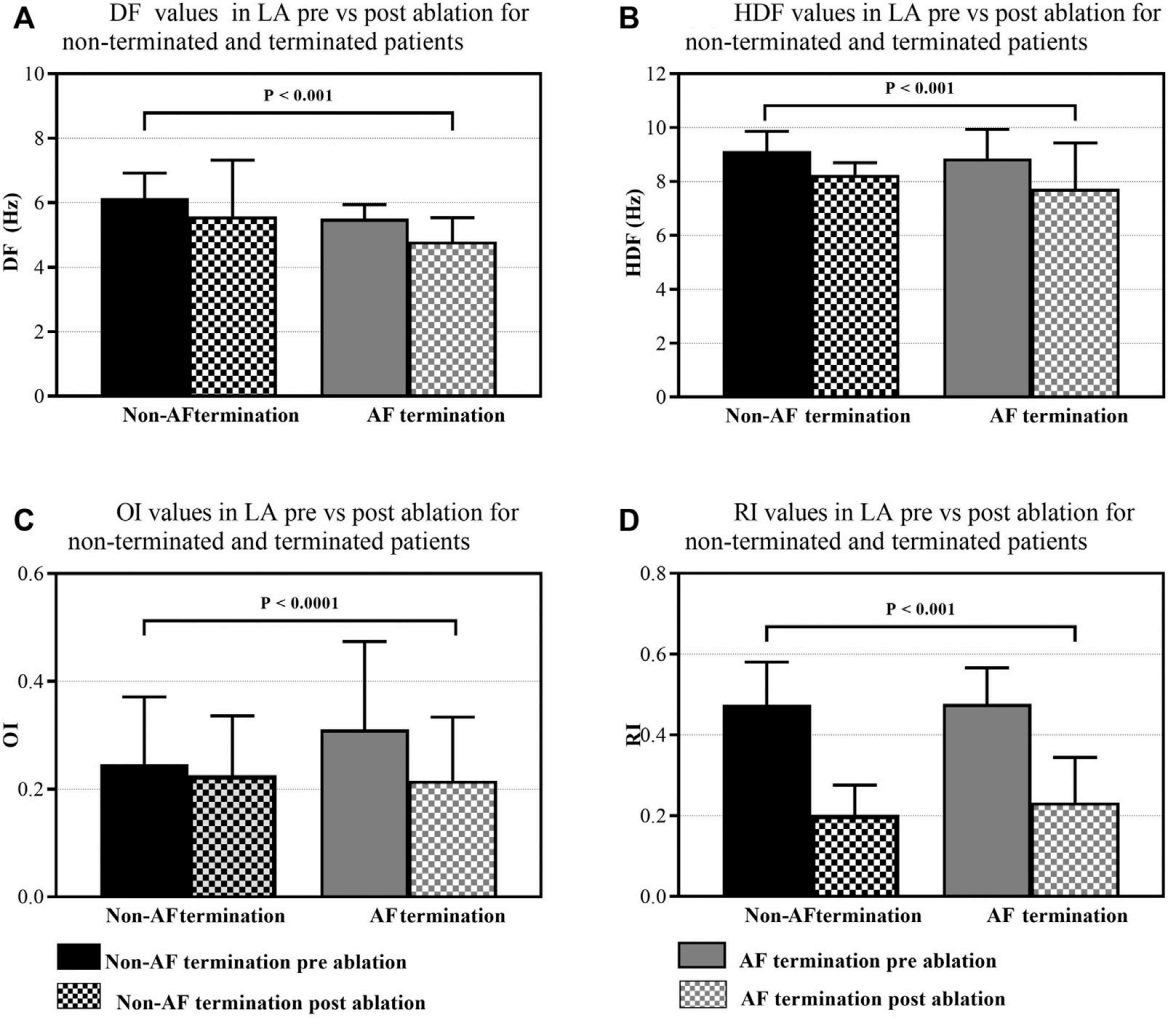
Monitoring the effect of HDF-guided ablation in PersAF patients. Bars represent the mean ± SD for each group. **(A)** DF across LA, pre-versus post-ablation for non-terminated and terminated patients. **(B)** HDF values change across LA, pre-versus post-ablation for non-terminated and terminated patients. **(C)** OI values change across LA, pre-versus post-ablation for non-terminated and terminated patients. **(D)** RI values change across LA, pre-versus post-ablation for non-terminated and terminated patients.

For the 3D geometry of the LA, the area of HDF regions (the 10% top decile of DFs over the 2048 points of the LA) were identified and a colour-scale HDF map was constructed for the LA. The creation of HDF density maps was produced by counting those HDF occurrences for every LA node. Therefore, all atrial areas with HDF were accordingly delineated for all frames regarding each of the 2048 AEG. A recording was made of the number of occasions that the HDF clouds superimposed on another. To achieve this a representation of the 3D LA geometry was used. Such HDF regions are thought of as representative of those locations whereby PersAF arrhythmia is maintained. Accordingly, an HDF “cloud” is formed by the HDF area that is presumed to represent the region’s AF activity. An automated algorithm was used to reconstruct the 2D (Figures 2B,D) and 3D HDF maps for (Figures 5C,D,G,H).

### Phase mapping of atrial electrograms

Phase analysis is a tool employed to track the progression of the electrical activation of a region of the myocardium (Umapathy et al., 2010). During the AF phase mapping, when analysing spatiotemporal phase maps, there is particular interest regarding points at which the phase goes through an entire cycle (from–π to +π). These are referred to as “phase singularities” PSs. The activation front thereby rotates around the PSs. Several methodological stages are involved in this phase analysis when converting the AEG into a phase map. The sinusoidal decomposition approach was used for the current study, using the following steps (Kuklik et al., 2016).

1). In the “sinusoidal decomposition method” used to recompose the atrial AEG, the representation of the signal is given as the total sum of the sinusoidal wavelets using a frequency that equals the DF and by employing an amplitude that corresponds (is proportional to) the atrial AEG negative gradient. 2) As part of this pre-processing process, a sinusoidal signal is generated that is suited to phase mapping utilising the Hilbert Transform. 3). Phase maps are created by calculating the instantaneous phase of each of the 2048 AEG channels Figure 1D. The phase of all the AEG can be assessed at every point in time over the entire surface of the recordings, thus enabling the construction of the “movie” of the evolution of the phase map. During organised activity, distinct phase patterns will be identified, such as PSs, which are automatically identified using an algorithm based on the topological charge method by (Bray et al., 2001) at the points where the phase progresses through a full cycle (from—π to + π). We tracked the recurrence of PSs with consecutive phase frames with a spatial distance of five nodes or less between consecutive frames. A rotor was defined as a series of PSs lasting over 100 ms and detected at a similar location (5 nodes or less) through subsequent time frames. As described in (Li et al., 2020), rotor velocity was calculated as the cumulative 3D geometry surface distance divided by the time, whilst rotor displacement was calculated as the surface distance between locations of the rotor appearing and disappearing.

### Assessing the spatial correlation between the density maps of rotors’ sites and high dominant frequency regions

In order to investigate the spatial correlation between the HDF regions and the sites of rotors density maps, we initially projected both HDF regions and rotors sites on 64 × 32 2D rectangles. The density information was removed to help assess the overlapping between the 2D maps. Figure 2B and D illustrate the 2D binary maps of the HDF regions and rotors’ sites, where the AEG of 2048 virtual electrodes were mapped onto a 64 × 32 2D rectangular grid, as described in (Li et al., 2020), HDF regions and rotors’ sites were marked as “ones” in a 64 × 32 matrix when they appear and marked as “zeros” where there were no HDF values or rotors at that specific “pixel.” The similarity of each 2D HDF binary maps and rotors’ binary maps were therefore measured using a 2D Pearson’s correlation coefficient (CORR) (Pearson, 1897), similarity index (Eq. 1) where A, B represent the 2D matrices; A̅, B̅ represent their respective average values, and i and j are the rows and columns of the 2D matrices.

$$CORR=\;\frac{\sum \left(A_{ij}-\bar{A}\right)\left(B_{ij}-\bar{B}\right)}{\sqrt{\sum {\left(A_{ij}-\bar{A}\right)}^{2}{\left(B_{ij}-\bar{B}\right)}^{2}}}$$ (1)

Secondly, to generate high-density maps of 3D LA geometry; the 64 electrodes on the non-contact MEA catheter are used to estimate AEG in 64 locations on the endocardium, which are further interpolated to provide a total of 2048 AEG, each AEG represented with a node on 3D LA geometry as described in (Li et al., 2020). Next, a colour-scaled LA 3D geometry was constructed, with HDF regions and rotors’ sites distributed across the LA. Each node out of the 2048 nodes that construct the 3D LA geometry was evaluated to determine whether or not it contains a value for either HDF regions or rotor sites or not at each time. The colour gradient of the LA geometries indicates the most visited region as red, the less often visited as yellow and the regions not visited at all as blue. IN these maps the ablated areas are marked with light red circles. Finally, up to 12 anatomical regions of the LA were manually marked on the LA geometries (where visible) by expert clinicians using the EnSite Velocity mapping system. Each anatomical region was marked with multiple surface points to create a closed representation of boundary points. The point number and its coordinates were saved and exported for off-line reconstruction showing the following: Roof; Septum (SEPT); MV isthmus (MVI); LAA; Anterior (ANT); and Posterior (POS); Floor; Right lower PVs (RLPV); Right upper PVs (RUPV); Left lower PVs (LLPV); Left upper PVs (LUPV); and Mitral Valve (MV).

This methodology enables the calculation of the spatial correlation between the rotors’ sites and HDF regions across the entire LA in both 2D and 3D scales. HDF and rotors’ density maps produced from individual 60 s of AEG recordings may highlight persistently dense regions and assist in identifying the individual drivers, which sustain PersAF. Therefore, to understand the co-localisation of rotors sites and the HDF region, we produced a combined map of cumulative rotors within the HDF region Figures 2B,D. This technique enabled the quantitative analysis of the overlap between the HDF regions and the rotors sites (or their non co-localisation) in a combined map. Furthermore, we projected the atrial areas that were ablated (red dots) on the same 2D combined map and 3D LA geometry to evaluate the effects of ablation of the HDF region in the non-termination patients and the termination group. A direct comparison of the pre- and post-ablation maps of HDF regions with rotor maps was carried out in both 2D and 3D maps to assess the effect of the ablation of HDF regions and the spatial distribution of rotors sites for both terminated and non-terminated patients. The spatial correlation between the sites of the rotors and the sites where the AFCL changed and led to AF termination was assessed by high-density maps in both 2D and 3D. Because all nodes associated with AFCL changes and rotors can be projected on 2D and 3D maps, the percentage of spatial overlapping was calculated using an algorithm that automatically reported the percentage of total rotors that coincide with AFCL change sites with respect to the total size of sites where there was AFCL change.

## Statistical analysis

All continuous variables with normal distribution were presented as (mean ± standard deviation). Wilcoxon matched-pairs signed-rank tests were performed to analyse non-parametric paired multiple data. Non-parametric unpaired data were analysed by means of the Mann—Whitney test. The pre- and post-ablation of the regional distribution of the sites of the rotors and HDF regions across the LA were compared using ordinary one-way ANOVA. A value of *p* < 0.05 was considered statistically significant.

## Results

### Baseline characteristics

This study achieved a 40% level of AF termination. A total number of lesion nodes (1,500, *N* = 4) in termination patients were ablated and (1970, *N* = 6) in non-termination patients. A total of 40,960 AEG and 80 sequential density maps of HDF regions and rotors’ sites were analysed to assess the effect of catheter ablation on their spatial stability. The results exhibited a very poor spatial correlation between the HDF regions and rotors sites in pre-versus post-ablation for both the non-terminated (pre: CORR; 0.0525 ± 0.17. vs. post: CORR; −0.0302 ± 0.19, Figure 2A, and with terminated patients (pre: CORR; −0.016 ± 0.03. post: CORR; −0.022 ± 0.04, Figure 2C). Figures 2B,D reveal a very low similarity between the HDF and rotors’ maps in non-terminated and terminated patients pre-and post-ablation cases.

### Monitoring the effect of left atrium high dominant frequency regions guided ablation in patients with PersAF

HDF-guided ablation prior to PVI has contributed to a slower atrial arrhythmia by decreasing the DF across LA for both groups, non-terminated and terminated patients (pre: 6.14 ± 0.7 Hz, vs. 5.50 ± 0.4 Hz, *p* < 0.001. vs. post: 5.57 ± 1.7 Hz, vs. 4.78 ± 0.7 Hz, *p* < 0.001, Figure 3A). The DF reduction was followed by a slight decrease in global HDF values (Figure 6A), for non-terminated and terminated patients (pre: 9.13 ± 0.7 Hz, vs. 8.85 ± 1.0 Hz, *p* < 0.001. vs. post: 8.25 ± 1.0 Hz, vs. 7.73 ± 0.7 Hz, Figure 3B). Additionally, the frequency organisation and regularity of the AEG of HDF regions as measured with OI and RI demonstrated that OI was significantly higher in terminated patients compared to non-terminated patients (pre: 0.31 ± 0.16 vs*.* 0.24 ± 0.12, *p* < 0.001, vs. post: 0.21 ± 0.11 vs*.* 0.22 ± 0.11, *p* < 0.0001 Figure 3C). Also there was statistical significance difference in RI values between non-terminated and terminated patients (pre: 0.47 ± 0.10 vs*.* 0.47 ± 0.11, vs. post 0.20 ± 0.07 vs. 0.23 ± 0.11, *p* < 0.001, Figure 3D).

### The effect of left atrium high dominant frequency-Guided ablation on rotor characteristics

**FIGURE 4:**
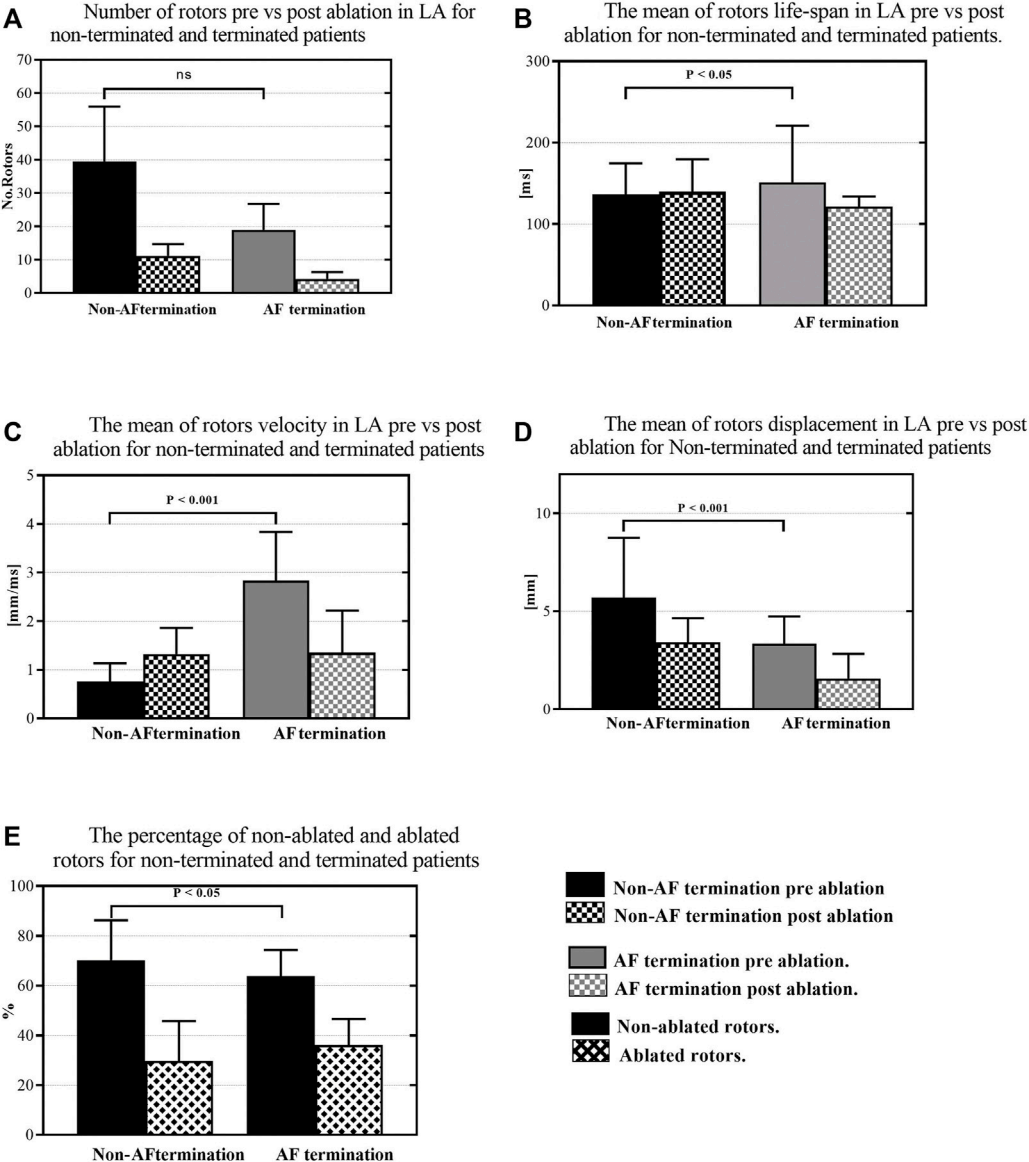
The response of the rotors to HDF ablation; **(A)** The number of rotors in pre-versus post-ablation for non-terminated and terminated patients. **(B)** The mean of the rotors life-span in pre-versus post-ablation for non-terminated and terminated patients. **(C)** The mean of the rotors’ velocity in pre-versus post-ablation for non-terminated and terminated patients. **(D)** The mean of the rotor displacement in pre-versus post-ablation for non-terminated and terminated patients. **(E)** The percentage of overall non-ablated and ablated rotors in non-terminated and terminated patients.

**FIGURE 5:**
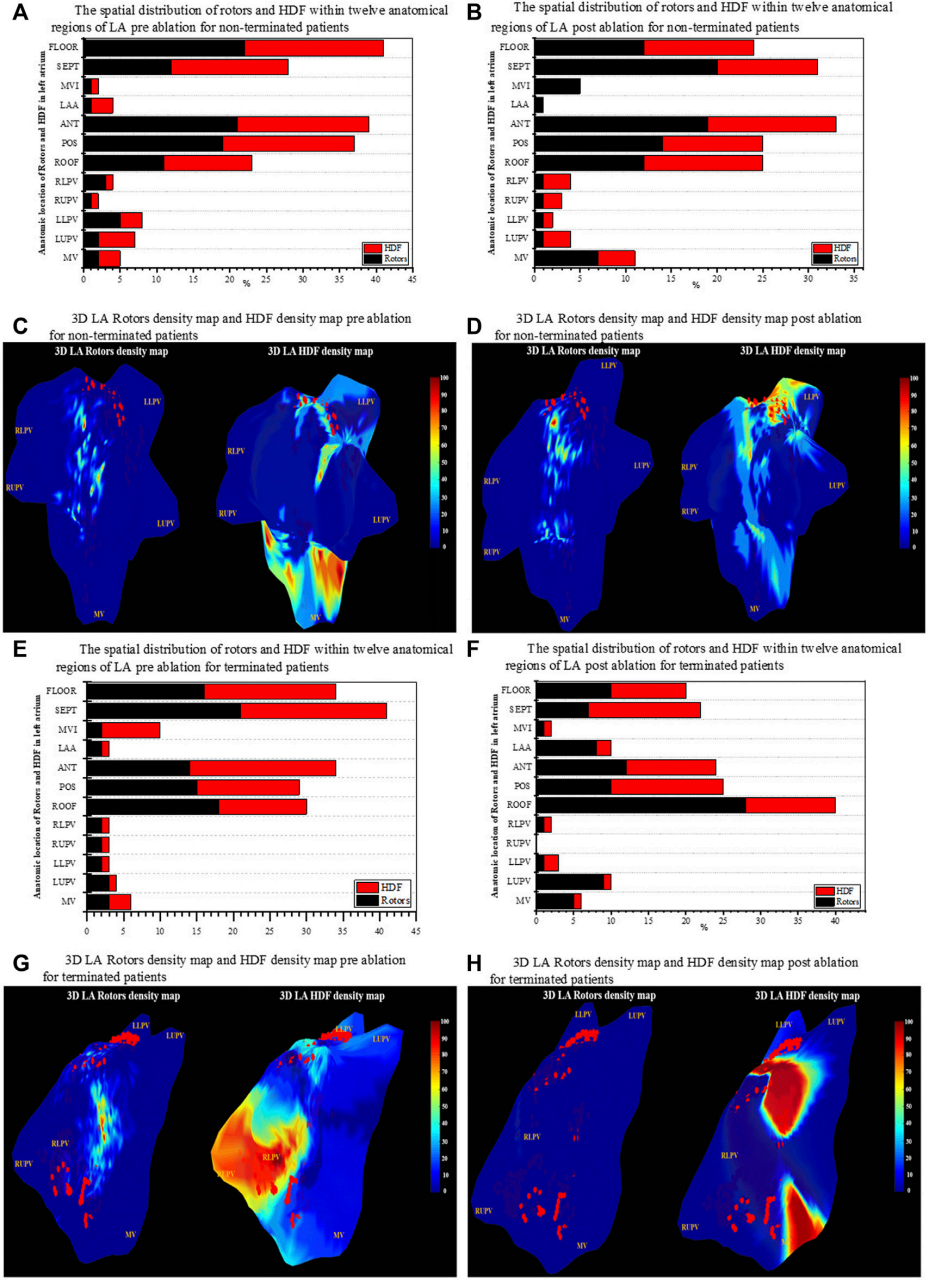
Regional distribution of rotors and HDF across the LA region. **(A)** Spatial distribution of HDF and rotors for 12 anatomical regions in LA for non-terminated patient pre-ablation. **(B)** Spatial distribution of HDF and rotors for 12 anatomical regions in LA for non-terminated patients post-ablation. **(C)** 3D LA geometry of rotors density and map HDF density map for non-terminated patient pre ablation. **(D)** 3D LA geometry of rotors density map and HDF density map for non-terminated patients post-ablation. **(E)** Spatial distribution of HDF and rotors for 12 anatomical regions in the LA for terminated patients pre ablation. **(F)** Spatial distribution of HDF and rotors for 12 anatomical regions in the LA for terminated patients post-ablation. **(G)** 3D LA geometry of rotors density map and HDF density map for terminated patients pre-ablation. **(H)** 3D LA geometry of rotors and density map HDF density map for terminated patients post-ablation. The colour bars in **(C,D,G,H)** represent the percentage of rotors sites and HDF regions on the 3D LA geometry. The red dots on the surface of the geometry represent the ablation lesions marked by the mapping system during the procedure.

**FIGURE 6:**
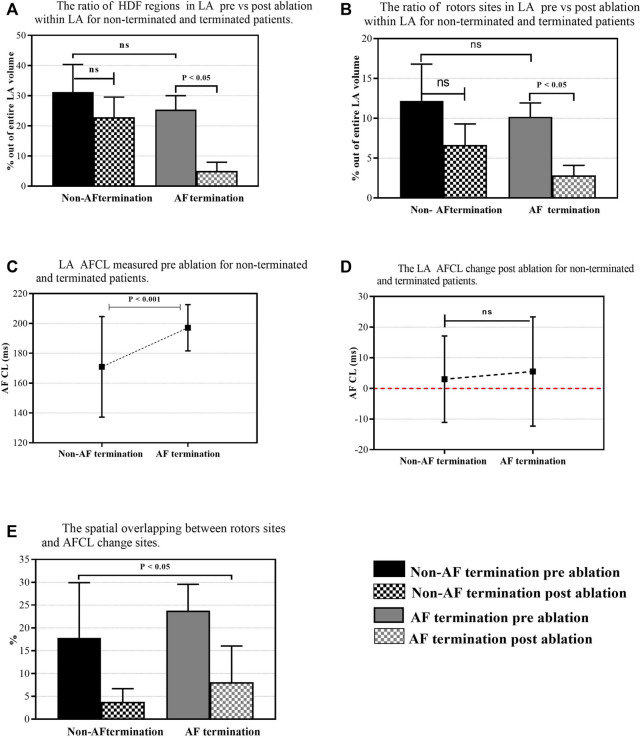
The overall percentage of rotors’ sites and HDF regions out of the entire LA area. **(A)** The ratio of the rotors’ sites through the LA pre-versus post-ablation for non-terminated and terminated patients. **(B)** The ratio of the HDF regions through the LA pre-versus post-ablation for non-terminated and terminated patients. **(C)** LA AFCL measured pre-ablation for non-terminated and terminated patients. **(D)** LA AFCL change was measured post-ablation for non-terminated and terminated patients. **(E)** The spatial overlapping between rotors’ sites and AFCL change sites.

The average number of detected rotors in non-terminated patients was higher than the terminated patients (pre: 39.50 ± 16.2 vs. 19 ± 15.6, vs. post: 11.7 ± 8.1 vs. 4.2 ± 2.4, Figure 4A). Thus, HDF guided ablation significantly affects the number of identified rotors in terminated patients, with a significant reduction (non-terminated patients vs. terminated patients: 29.7 ± 5.2%. vs. 36.1 ± 5.5%, Figure 6B). In contrast, the life-span of the tracked rotors was higher in the terminated group compared to the non-terminated patients prior to ablation (pre: 151.3 ± 69.51 ms vs. 136.4 ± 38.2 ms vs. post: 120.7 ± 6.5 ms vs. 139.9 ± 39.8 ms, Figure 4B). Likewise, rotor core velocity was higher in terminated patients than non-terminated group (pre: 2.8 ± 1.9 mm/ms vs. 0.7 ± 0.9 mm/ms vs. post: 1.35 ± 1.3 mm/ms vs. 1.32 ± 0.9 mm/ms, Figure 4C). However, rotor core displacement was higher in non-terminated compared to terminated patients (pre: 5.6 ± 7.4 mm vs. 3.34 ± 2.7 mm vs. post: 3.4 ± 3.04 mm vs. 1.5 ± 1.2 mm, Figure 4D). Figure 4E denotes the percentage of rotors not ablated (non-terminated patients vs. terminated patients: 70.2% ± 6.5 *Vs.* 63.1% ± 5.4). Figures 5C,D,G,H show that detected stable rotors of AEG lack spatial stability over pre-and post-ablation.

### Spatial distribution of high dominant frequency regions and rotors’ sites through the left atrium

Despite the fact of AF termination, Figures 5A,B,E,F showed that HDF regions and rotors’ sites appear to have a relationship with the most visited anatomical regions of LA for pre and post-ablation. Interestingly the septum is the most visited region—non-terminated patients vs. terminated patients (mean ± SD pre: 41 ± 12.4%. vs. post: 34 ± 9.07%), followed by floor (33 ± 2.30%. vs. post: 30 ± 1.4%), anterior site (29 ± 1.31%. vs. post: 16 ± 2.3%), posterior site (25% ± 1.5. vs. post: 11 ± 0.5%) and roof (18 ± 0.23%. vs. post: 9 ± 0.13%) respectively. Therefore, in 60 s segments of PersAF recordings, the preferential areas visited by the HDF and Rotors were the septum and the floor of the LA, with the highest incidence of PSs compared with the remaining LA areas. RUPV and the anterior wall of the LA presented almost half the incidences of PSs. Figures 5C,D,G,H illustrate the results of the projected HDF regions and rotors’ sites on 3D geometries of LA’s surface for non-terminated and terminated patients. Relying on a visual inspection, it is clear that rotor sites are localised predominantly around or at the vicinity of HDF regions in both groups. In non-terminated patients, although the dense HDF region locations were targeted, both rotors focal sites and HDF regions were widely dispersed all over the surface of the LA post-ablation. Conversely, with a terminated patient where there was some spatial overlapping between rotors’ sites and the targeted HDF region prior to ablation, rotors seemed to have responded to ablation. Moreover, post-ablation density maps appeared free of rotors in this particular patient who converted to SR.

### Change in AF cycle length during ablation

For ten patients a total of 3,206 nodes were ablated: AFCL increase (≥10 ms, 947 nodes, 30%), AFCL non-increase (<10 ms, 2,259 nodes, 70%). Figure 6C shows the results of the AFCL measured at all ablation sites pre-ablation for non-terminated and terminated patients (Mean ± SD 170.9 ± 33.7 ms vs. 197.1 ± 15.5 ms, *p* < 0.001, respectively), where the mean of AFCL per patient for non-terminated and terminated pre ablation is (Mean ± SD 99.7 ± 35.4 ms vs. 78.8 ± 12.7 ms), However, the response of the ablation of the prolongation in AFCL during the procedure was not significant between the two patients groups; post-ablation: AF non-terminated patients vs. AF terminated Figure 6D (Mean ± SD 3 ± 14 ms, vs. 5.5 ± 17.8 ms). Although the results suggest a poor spatial correlation between rotors’ sites and sites of the AFCL changes in terminated and non-terminated patients, a higher correlation was found in terminated patients (spatial overlapping percentage pre: 25 ± 4.2% vs. 17 ± 3.8% vs. post: 8 ± 4.2% vs. 3.7 ± 1.7% *p* < 0.05, respectively, Figure 6E).

## Discussion

This study provides further insight into the influence of HDF ablation on the spatial stability of HDF regions and rotors’ sites. For all patients, the NC-AEG shows that rotors’ sites are poorly spatially correlated with HDF regions. High-density maps, HDF and rotors sites appear to have a relationship with LA’s most visited anatomical regions for both pre-and post-ablation, interestingly with the septum being the most visited region. HDF guided ablation significantly affects the number of tracked rotors, particularly with a significant reduction in those where AF was terminated. Rotor sites detected in terminated patients had specific characteristics. They had a long-lasting life-span and high core velocity, and they were less meandering than sites detected in non-terminated patients. The rotors’ sites demonstrated poor simultaneous spatial overlapping of the location of the rotors and the sites of AFCL changes that lead to AF termination.

### Analysing spatial disparities of high dominant frequency regions and rotors’ sites across the left atrium

Multiple studies have reported contradictory results regarding the spatial distribution of HDF regions in PersAF patients across LA. Elvan et al. (2009) found that HDF sites are predominantly located in the posterior wall between the pulmonary veins and at the roof across the LA. Sanders et al. (2005), reported the HDF regions are localised on other non-PV regions of the LA and Suenari et al. (2011), reported that the highest DF were primarily located inside the PVs ostia. Conversely, our results showed that HDF dense regions seemed to have the highest incidence at the septum followed by the floor, anterior wall, and posterior wall of the LA and floor, respectively.

On the other hand, using non-contact mapping during human AF, Yamabe et al. (2016), reported that the rotor activation sites tend to be observed around the roof, septum and left superior PVs. Using a basket catheter, Swarup et al*.*, reported that stable rotors and focal sources lay in multiple locations across LA remote from the PV. The posterior wall was the most visited region followed by the roof, anterior wall, near left PVs, along with the near right side of the PVs. Our results show that rotors were observed with the highest incidence within the septum, followed by the floor, anterior wall, and the posterior wall of the LA, respectively.

Using 3D human AF models, Hwang et al. (2016), reported that the HDF regions were well-correlated with the rotor sites and ablating such HDF sites resulted in slow atrial tachycardia. Our results also suggest a hidden interaction between the rotors and HDF regions. We observed that the HDF regions and the sites of the rotors appear to have a relationship with the LA’s most visited anatomical regions for both pre and post-ablation of HDF regions, with the septum being the most visited region, followed by the floor anterior site, posterior site and roof. This interesting correlation could suggest a relationship between both tracked phenomena. The key questions remain: are these active drivers of fibrillation or just simply a passive phenomenon? And is this phenomenon a consequence of the other? Therefore, which is more likely to be the main driver of the overall arrhythmia? The answers remain ambiguous and require further investigation.

### Ablation response of targeting high dominant frequency regions

In the present study, ablating the HDF regions prior to PVI coincided with the termination of AF in 4 out of the 10 patients with one patient converting to SR and three patients converting to atrial flutter. In the other six AF patients, the arrhythmia did not terminate even after PVI. Interestingly, for the terminated AF patients, the ablation resulted in a reduction in DF gradient throughout LA. This result is consistent with the findings that suggest targeting the HDF reduces the DF of AF electrograms (Lemola et al., 2006; Takahashi et al., 2006; Tuan et al., 2011) and is associated with an improvement in the ablation outcome (Yoshida et al., 2010). The reduction in DF could be explained as a result of decrease in overall atrial activation rates (Yoshida et al., 2010).

Interestingly, our study also found that OIs were significantly higher when compared between terminated vs. non-terminated AF patients. This finding agrees with previous studies that reported that an increase in OI prior to ablation can predict sites of AF termination (Tuan et al., 2010).

The difference in RI values were also statistically significant among the patient groups prior to ablation. Kalifa et al. (2006) reported that the highest RI regions coincide with the region of HDF region and vice versa. Lin et al. (2016) implied that higher RI values means that the propagation of the waves rotates around the rotor. For all patients, our results showed that the average RI values were higher than 0.4 prior to ablation, and significantly reduced to 0.2 post-ablation. This reduction could be due to the shape of the impinging wavefront at the boundaries of the HDF regions. Our finding agrees with studies stating that DF sites selected by an RI threshold higher than 0.2 are potential candidates for ablation (Traykov et al., 2012).

### Rotors response during catheter ablation for PersAF targeting high dominant frequency regions

Literature reveals contradictory results regarding identifying rotor sites during AF mapping using a non-contacting cardiac mapping system. Yamabe et al. (2016), rotor sites were present in only one patient out of the 15 PersAF patients enrolled. According to them, rotors may not be the primary source of perpetuating AF. Lee et al., 2015 have failed to detect rotors or any sustained focal sources in 15 PersAF patients. Contrastingly, in this study using NC-AEG, rotors were observed in all of the 10 recruited patients. Thus, our results are consistent with the findings of (Kanemaru et al., 2016; Kaneko et al., 2019). Many studies have demonstrated the importance of rotor parameters, for instance, rotor life-span, rotor conduction velocity and rotor core displacement (or “rotor meandering”) to reflect the spatiotemporal stability of rotors during AF mapping (Hwang et al., 2016; Lim et al., 2017). Lim et al. (2017) reported that in Virtual ablation, using a homogeneous monolayer computer model targeting a high DF area, the successful rate of AF terminations was depended on rotors parameters, where AF easily terminated spontaneously with a lower number of rotors prior to ablation and rotors with a shorter rotors life-span and a certain conduction velocity of ≥0.5 m/s. Nonetheless, our results confirm that rotor sites detected in terminated patients had specific characteristics. It was observed that they have a long-lasting life-span and high core velocity, and they were less meandering compared to sites detected in non-terminated patients. Thereby, rotor sites play an important role in driving or maintaining the AF with PersAF patients. The change in rotor parameters could be explained as a result of the fibrillation complexity in PersAF patients that is related to an increase of spontaneous cell activation in cardiac tissue and atrial heterogeneity. This might be a consequence of multiple factors, such as ion channel expressions and higher degrees of fibrosis in cardiac tissues (Ashihara et al., 2012; Pandit and Jalife, 2013; Climent et al., 2015). These phenomena affect the rotor formation characteristics of the rotors’ parameters. Thus, further studies are necessary to understand the effect of different phenomena, such as ionic basis and fibrosis, on rotors’ dynamics behaviour.

### Relationship between the rotors’ sites and the AF cycle prolongation sites

To improve the outcomes of catheter ablation and increase the predictability of the long-term freedom of PersAF, many studies have investigated the correlation between AF drivers’ sites and the site where the AFCL prolongation has occurred at which the AF terminate (Haïssaguerre et al., 2004; Kochhäuser et al., 2017). Honarbakhsh et al. (2018b), used a CARTO Finder mapping system with basket catheter targeting rotational or focal activity drivers for PersAF patients and reported a strong correlation between rotational or focal drivers and fastest AFCL. However, our study has shown that when using NC-AEG during HDF-guided ablation for ten PersAF patients, the rotors’ sites demonstrated poor simultaneous spatial overlapping of the location of the rotors and the sites of AFCL changes that lead to AF termination. This discrepancy could be explained because the spatial distribution of the PersAF drivers across the LA may vary according to individual characteristics and arrhythmia durations.

## Limitations of the study

This study was conducted with a relatively small sample size (*N* = 10). As our main aim was to investigate the spatial distributions of the HDF regions and rotor sites using high-density NC-AEG in PersAF, all NC-AEG analysis was restricted to the LA, and the right atrium was not studied here. Also, all analyses were performed retrospectively.

Despite the obvious advantage of providing AEG for the entire chamber via non-contact mapping, there are some limits. Virtual unipolar electrograms created using non-contact electrical mapping were reported to be vulnerable to inaccuracies during the AF recordings. These might be the result of: 1) potential errors in the inverse problem solution (Nakagawa et al., 2019); 2) inaccurate reconstruction of endocardial geometry of LA (Earley et al., 2006); 3) the assumption of electrical isotropic properties of the blood in the cardiac chambers (Meng et al., 2022); and 4) aliasing due to the limited spatial sampling (Zhao et al., 2013). These constraints may also have an influence on defining NC-AEG properties, such as DF and PSs, when compared to contact AEG during AF recordings.

## Conclusion

The complex dynamics of high-frequency regions characterised by HDF were poorly spatially correlated with rotors’ sites. However, the HDF regions and rotors do visit the same anatomical regions of LA, with the septum being the most visited region followed by the floor, anterior site, posterior site and roof, respectively. Thus, this finding supports the theory that the AF is initiated and maintained by multiple mechanisms across LA. Our results have demonstrated the consistency of using NC-AEG to investigate the re-entrant activity across the LA for PersAF patients. Nevertheless, we discovered that HDF-guided ablation does affect the characteristics of the rotors’ sites in those patients where AF was terminated. Remarkably, rotors associated with AF terminations were distinct with particular parameters; they had a long-lasting life-span, high core velocity, and were less meandering across LA compared to sites detected in non-terminated patients. Thus not all rotors’ sites are promising targets for PersAF ablation. Using NC-AEG, the sites of the rotors demonstrated poor (simultaneous) spatial overlapping with the sites of AFCL changes that lead to AF termination. This suggests that if there is an interaction between these two PersAF attributes, the dynamics of such interaction is more complex than a simple simultaneous spatial correlation.

## Data Availability

The original contributions presented in the study are included in the article/supplementary material, further inquiries can be directed to the corresponding author.
